# Increased Oxygen Desaturation Time During Sleep Is a Risk Factor for NASH in Patients With Obstructive Sleep Apnea: A Prospective Cohort Study

**DOI:** 10.3389/fmed.2022.808417

**Published:** 2022-02-23

**Authors:** Pedro Landete, Carlos Ernesto Fernández-García, Beatriz Aldave-Orzaiz, Marta Hernández-Olivo, Carmen M. Acosta-Gutiérrez, Enrique Zamora-García, Julio Ancochea, Águeda González-Rodríguez, Carmelo García-Monzón

**Affiliations:** ^1^Servicio de Neumología, Hospital Universitario de La Princesa, Instituto de Investigación Sanitaria del Hospital Universitario de La Princesa, Madrid, Spain; ^2^Departamento de Medicina, Universidad Autónoma de Madrid, Madrid, Spain; ^3^Unidad de Investigación, Hospital Universitario Santa Cristina, Instituto de Investigación Sanitaria del Hospital Universitario de La Princesa, Madrid, Spain; ^4^Centro de Investigación Biomédica en Red de Diabetes y Enfermedades Metabólicas Asociadas (CIBERDEM), Madrid, Spain

**Keywords:** non-alcoholic fatty liver disease, steatosis, non-alcoholic steatohepatitis, obstructive sleep apnea syndrome, nocturnal intermittent hypoxia

## Abstract

**Introduction:**

Given that obstructive sleep apnea (OSA) is commonly associated with metabolic disorders, in this prospective study, we sought to determine the prevalence and risk factors for hepatosteatosis, non-alcoholic steatohepatitis (NASH), and advanced liver fibrosis in patients with clinical and polygraphic criteria of OSA (*n* = 153) and in subjects with normal lung function parameters (NLP, *n* = 43).

**Methods:**

Hepatosteatosis, NASH, and advanced liver fibrosis were determined by blood-based non-invasive tools, such as the fatty liver index and the hepatic steatosis index, a serum lipidomic (OWLiver™) test, and three distinct fibrosis algorithms, respectively. Logistic regression models adjusted by potential confounders were performed to evaluate risk factors.

**Results:**

Insulin resistance and dyslipidemia were more frequent in patients with OSA than in subjects with NLP. The prevalence of hepatosteatosis was significantly higher in patients with OSA than in subjects with NLP. NASH was also found more frequently in patients with OSA than in subjects with NLP. In contrast, advanced liver fibrosis was rarely detected in the entire study population, and no significant differences were observed between patients with OSA and subjects with NLP. Besides male gender, increased body mass index (BMI), and presence of type 2 diabetes, percentage of sleep time with oxygen saturation <90% (Tc90%) was the only polygraphic variable significantly associated with NASH in patients with OSA.

**Conclusions:**

This study shows that hepatosteatosis and NASH are highly prevalent in patients with OSA and indicates that those with a Tc90% higher than 10% are at increased risk for NASH.

## Introduction

Obstructive sleep apnea (OSA), a chronic respiratory disorder featured by nocturnal intermittent hypoxia, is commonly associated with a number of metabolic disorders such as obesity, type 2 diabetes (T2D), and non-alcoholic fatty liver disease (NAFLD) (1–5). The latter comprises a range of liver histopathological alterations from hepatosteatosis, mostly a benign non-progressive clinical entity defined as the presence of fat in >5% of hepatocytes, to non-alcoholic steatohepatitis (NASH), a more severe condition featured by steatosis, lobular and portal inflammation, and degeneration (ballooning) of hepatocytes with or without fibrosis, which in turn can lead to more severe conditions of liver disease such as cirrhosis and hepatocellular carcinoma (HCC) (6).

There is an increasingly experimental and clinical evidence indicating that a pathophysiological link between nocturnal intermittent hypoxia and NAFLD progression exists (7–18). Given that the global burden of both OSA and NAFLD is steadily growing (19, 20), it is of paramount importance to increase the awareness of these comorbidities among physicians caring for at-risk populations. In that regard, it is well-known that NASH is a fibrogenic disorder, and advanced liver fibrosis is the major determinant of liver-related and all-cause morbidity and mortality of patients with NAFLD (21, 22). Thus, the implementation of strategies facilitating the early identification of patients at high risk of NASH and/or advanced liver fibrosis must be a crucial goal for public health systems.

Although liver biopsy is currently considered as the gold standard method for NASH diagnosis and fibrosis staging in patients with NAFLD, its use is impractical and even unethical for large observational clinical studies. To that end, distinct blood-based, non-invasive, and easy-to-use scores have been developed to predict hepatosteatosis with a good performance, such as the fatty liver index (FLI) (23) and the hepatic steatosis index (HSI) (24), and to identify patients with significant liver fibrosis, such as FIB-4 (25), NAFLD fibrosis score (NFS) (26), and Hepamet fibrosis score (HFS) (27). In addition, the commercially-available OWLiver™ test is a serum-based lipidomic assay that discriminates hepatosteatosis from NASH with high accuracy and has been validated in clinical studies using blind-histology assessment of liver biopsy-proven patients with NAFLD (28, 29).

Therefore, in this study, we sought to determine, by using a wide panel of blood-based non-invasive tools, the prevalence of and risk factors for NAFLD (hepatosteatosis and NASH) and advanced liver fibrosis in patients with OSA, searching for potential associations with baseline clinical and analytical and polygraphic features of the study population.

## Materials and Methods

### Study Population

This prospective cross-sectional cohort study included consecutive patients with clinical and polygraphic criteria of OSA among those who attended the outpatient clinics of the Pneumology Service at Hospital Universitario de La Princesa (Madrid, Spain) during a 3-month period. In parallel, volunteers who had sleep polygraphy within normality were included in the study and considered as controls (subjects with normal lung function parameters, NLP). Patients and controls were excluded if they drank more than 20 g/day of alcohol, had a diagnosis of asthma or cancer, or any concomitant severe clinical disorder. In addition, they were also excluded if they had analytical evidence of iron overload (transferrin saturation index 55%), were seropositive for autoantibodies, and/or for hepatitis B virus (HBV), hepatitis C virus (HCV), HIV, and/or had used potentially hepatotoxic drugs.

This study was performed in agreement with the Declaration of Helsinki, and with local and national laws. The Clinical Research Ethics Committee of the Institution (Instituto de Investigación Sanitaria del Hospital Universitario de La Princesa, Madrid, Spain) approved the study procedures (Report reference: PI/2800-16), and all participants signed informed written consent before inclusion in the study, providing permission for their medical data to be anonymously used for research.

### Clinical and Laboratory Assessment

Clinical examination was performed on all participants in this study, including a detailed interview with special emphasis on smoking pattern, alcohol intake and medication use, history of diabetes, and arterial hypertension, as well as measurements of weight, and height. Body mass index (BMI) was calculated as weight (kilograms) divided by height (meters) squared. A BMI ≥ 30 kg/m^2^ was defined as obesity. After a 12-h overnight fast, venous blood samples of each participant were obtained to test serum levels of liver enzymes, metabolic parameters, and autoantibodies using routine laboratory methods. Blood-based algorithms were used to predict hepatic steatosis, FLI (23) and HSI (24), and the absence or presence of liver fibrosis by using FIB-4 (25), NFS (26), and HFS (27). Serum lipidomic profiling was determined for all patients in the study and control subjects by using the OWLiver™ test (One Way Liver SL, Derio, Spain), a commercially-available assay recently validated to distinguish simple steatosis and NASH with excellent diagnostic accuracy (28). In addition, plasma insulin was determined by a chemiluminescent microparticle immunoassay (ARCHITECT insulin; Abbot Park, IL). Insulin resistance was calculated by the homeostasis model assessment (HOMA-IR) method (30). Metabolic syndrome was defined according to the Adult Treatment Panel II (ATP III) criteria (31). Antibodies against HCV, HIV, and HBV surface antigen were tested by immunoenzymatic assays (Murex, Dartford, UK).

### Cardiorespiratory Polygraphic Study

The vast majority of polygraphic studies were performed at night in the Sleep Laboratory of the Hospital Universitario de La Princesa. However, in some participants with limitations to hospital admission, the polygraphic studies were carried out at home by the usual caregivers. The parameters measured included airflow measurement with an oronasal thermistor and nasal pressure transducer; thoracoabdominal movement measured by impedance plethysmography; and pulse oximetry and microphone recording to evaluate snoring, breathing patterns, and movement. A previously validated cardiorespiratory polygraphy equipment (SOMNOscreen^TM^ Plus, Randersacker, Germany) with a Domino analysis software (Domino Data Lab, San Francisco, CA) was used. For interpretation, the recommendations of the American Academy of Sleep Medicine for OSA in adults were followed. The reading of the polygraphic record was made manually, although assisted by a computer. Apnea was defined as the absence of oronasal airflow and the absence of signal in the thermistor for more than 10 s, and hypopnea as the decrease in basal airflow in the mouth and nose between 30 and 90% accompanied by a significant desaturation (decrease in oxygen saturation >3% with respect to the previously recorded level). Episodes of apnea were further characterized as central or obstructive. Central apnea was defined as the absence of oronasal airflow and of thoracic and abdominal movements in the absence of bodily movements. The interruption of airflow in the nose and in the mouth, associated with movements of the thoracic cage and abdomen, was considered as obstructive apnea. The presence of an apnea and hypopnea index (AHI) ≥5 per hour was used as a diagnostic criterion for the certainty of OSA. The severity of OSA was classified according to the value of AHI as mild (AHI, 5–14/h), moderate (AHI, 15–29/h), or severe (AHI, ≥30/h). In addition, other polygraphic parameters were analyzed, such as the oxygen desaturation index (ODI), defined as the number of oxygen desaturations per hour during the whole sleep, and the percentage of sleep time with oxygen saturation below 90% (Tc90%). Both ODI and Tc90% were considered low when lower than 10 events/hour or lower than 10%, respectively, and were considered high when equal or higher than 10 events/hour and equal or higher than 10%, respectively.

### Statistical Analysis

The Kolmogorov-Smirnov test was applied to evaluate whether the variables were adjusted to a normal distribution. Qualitative variables are presented as absolute (number, *n*) and relative (percentage, %) frequencies. Quantitative variables are expressed as measures of central tendency (mean) and dispersion (standard deviation, SD). Qualitative data between groups were compared by Pearson's *c*^2^ -test or Fisher exact test as appropriate. Student's *t*-test was used to calculate the difference of the means in the variables that followed a normal distribution, and the Mann Whitney *U*-test was used for the variables with a non-parametric distribution. Logistic regression analysis, adjusted by confounding variables (age, gender BMI, and T2D), was performed to identify independent polygraphic variables (AHI, ODI, and Tc90%) associated with metabolic (T2D, insulin resistance, dyslipidemia, and metabolic syndrome) and liver outcomes (hepatosteatosis, NASH, and advanced liver fibrosis) in the study population. Univariate and multivariate regression models were constructed, and parameters were selected by the likelihood ratio test. The Box-Tidwell procedure was used for testing linearity of logit and to obtain a linear logit the appropriate transformation of variables was used. The goodness-of-fit of the model was evaluated using the Hosmer-Lemeshow statistic. Significance was set at a value of *p* < 0.05. Statistical analysis was performed using SPSS software version 26.0 (SPSS Statistics, Armonk, NY: IBM Corp).

## Results

### Characteristics of the Study Population

As shown in the flowchart depicted in Supplementary Figure 1, a total of 225 subjects were initially enrolled in this study. Five patients were subsequently excluded due to active cancer and eight due to asthma. We also excluded seven subjects who drank more than 20 g/day of alcohol and nine who did not perform the polygraphic study. Finally, a total of 196 subjects, including 153 patients with polygraphic features of OSA and 43 subjects with normal lung function parameters (NLP group), who fulfilled all inclusion and exclusion criteria were analyzed.

Demographic, anthropometric, and analytical characteristics of the entire study population are detailed in Table 1. Overall, men were predominant in the OSA group while the majority of individuals in the NLP group were women, and this was the only statistically significant difference between both the groups.

**Table 1:** Characteristics of the study population.

**Features**	**NLP**	**OSA**	***p*-value**
	**(*n* = 43)**	**(*n* = 153)**	
Age (years)	56.1 ± 8.9	59.8 ± 8.5	0.863
Gender			**0.001**
Women, *n* (%)	27 (62.8)	54 (35.3)	
Men, *n* (%)	16 (37.2)	99 (64.7)	
Body mass index (kg/m^2^)	28.6 ± 5.1	29.8 ± 5.8	0.297
Glucose (mg/dL)	100.4 ± 16.0	105.7 ± 29.2	0.322
Insulin levels (μU/L)	12.6 ± 7.3	16.6 ± 12.7	0.293
HOMA-IR score	3.2 ± 2.2	4.8 ± 7.2	0.130
Glycated Hb (%)	5.6 ± 0.5	5.7 ± 0.7	0.923
Triglycerides (mg/dL)	115.5 ± 67.2	135.6 ± 79.6	0.318
Total cholesterol (mg/dL)	200.3 ± 38.9	193.3 ± 36.5	0.589
HDL-cholesterol (mg/dL)	56.9 ± 13.0	55.1 ± 19.2	0.369
LDL cholesterol (mg/dL)	117.0 ± 32.1	110.5 ± 34.1	0.706
VLDL cholesterol (mg/dL)	23.2 ± 13.5	26.9 ± 15.1	0.467
ALT (IU/L)	22.7 ± 13.1	24.4 ± 13.2	0.321
AST (IU/L)	21.3 ± 6.5	23.1 ± 10.8	0.338
GGT (IU/L)	25.7 ± 15.8	36.9 ± 28.9	0.263
Alkaline phosphatase (IU/L)	69.8 ± 21.4	68.1 ± 19.4	0.211
Total bilirubin (mg/dL)	0.6 ± 0.4	0.6 ± 0.3	0.179
Albumin (g/dL)	4.4 ± 0.3	4.4 ± 0.3	0.394
Iron (μg/dL)	83.8 ± 34.5	88.8 ± 28.5	0.229
Ferritin (ng/mL)	87.4 ± 72.1	143.3 ± 98.5	0.494
Transferrin (mg/dL)	244.3 ± 31.6	251.8 ± 37.4	0.504
Platelets (10^9^/L)	230.9 ± 66.8	229.6 ± 51.9	0.542
C reactive protein (mg/L)	0.4 ± 0.5	0.4 ± 0.5	0.370

*Data are shown as mean ± standard deviation or as number of cases (%). NLP, subjects with normal lung parameters; OSA, obstructive sleep apnea; HOMA-IR, homeostatic model assessment-insulin resistance; Hb, hemoglobin; HDL, high-density lipoprotein; LDL, low-density lipoprotein; VLDL, very low-density lipoprotein; ALT, alanine aminotransferase; AST, aspartate aminotransferase; GGT, gamma-glutamyltransferase. Bold values are the statistically significant*.

Taking into account the pulmonary function parameters, as expected, patients with OSA had a significantly higher result in both AHI and rate of oxygen desaturation per hour of sleep (ODI) and a higher percentage of sleep time with oxygen saturation lower than 90% (Tc90%) with respect to subjects with NLP, being these differences statistically significant (Table 2).

**Table 2:** Polygraphic features of the study population.

	**NLP**	**OSA**	***p*-value**
	**(*n* = 43)**	**(*n* = 153)**	
AHI (events/hour)			**<0.001**
<5, *n* (%)	43 (100)	0 (0)	
5–14, *n* (%)	0 (0)	55 (35.9)	
15–29, *n* (%)	0 (0)	46 (30.1)	
≥30, *n* (%)	0 (0)	52 (34.0)	
ODI (events/hour)			**<0.001**
<10, *n* (%)	42 (97.7)	25 (16.4)	
≥10, *n* (%)	1 (2.3)	128 (83.6)	
Tc90%			**<0.001**
<10%, *n* (%)	38 (88.4)	73 (47.7)	
≥10%, *n* (%)	5 (11.6)	80 (52.3)	

*Data are shown as mean ± standard deviation or as number of cases (%). NLP, subjects with normal lung function parameters; OSA, obstructive sleep apnea; AHI, apnea-hypopnea index; ODI, oxygen desaturation index; Tc90%, percentage of sleep time with oxygen saturation <90%*.

### Prevalence of Metabolic Disorders

Obesity (BMI ≥ 30), T2D, and metabolic syndrome were more frequent in patients with OSA than in subjects with NLP, but the differences were not statistically significant (Table 3). Notably, HOMA-IR values were higher in the OSA group than in controls, but these differences were not statistically significant as well (Table 1). However, insulin resistance, defined as a HOMA-IR > 3, was significantly more frequent in patients with OSA than in subjects with NLP (*p* = 0.001). Moreover, dyslipidemia was also significantly more common in patients with OSA than in subjects with NLP (*p* = 0.003) (Table 3).

**Table 3:** Metabolic and hepatic parameters of the study population.

**Features**	**NLP**	**OSA**	***p*-value**
Frequency, *n* (%)	43 (21.9)	153 (78.1)	NA
Body mass index ≥ 30, *n* (%)	13 (30.2)	68 (44.4)	0.095
HOMA-IR score ≥ 3, *n* (%)	14 (32.6)	93 (60.8)	**0.001**
Type 2 diabetes mellitus, *n* (%)	4 (9.3)	25 (16.3)	0.334
Dyslipidemia, *n* (%)	10 (23.3)	74 (48.4)	**0.003**
Metabolic syndrome, *n* (%)	4 (9.3)	33 (21.6)	0.080
**Hepatic steatosis**			
by Fatty Liver Index ≥ 60, *n* (%)	15 (34.9)	95 (62.1)	**0.001**
by Hepatic Steatosis Index ≥ 36, *n* (%)	21 (48.8)	104 (68)	**0.021**
NASH by OWLiver test, *n* (%)	12 (27.9)	77 (50.3)	**0.009**
**Low risk of advanced fibrosis**			
FIB-4 <1.3, *n* (%)	24 (55.8)	94 (61.4)	0.416
NFS < −1.455, *n* (%)	21 (48.8)	64 (41.8)	0.413
HFS <0.12, *n* (%)	35 (81.4)	121 (79.1)	0.833
**High risk of advanced fibrosis**			
FIB-4 > 3.25, *n* (%)	0 (0)	2 (1.3)	1.000
NFS > 0.675, *n* (%)	1 (2.3)	10 (6.5)	0.461
HFS ≥ 0.47, *n* (%)	1 (2.3)	4 (2.6)	1.000

*Data are shown as mean ± SD or as number of cases (%). NLP, subjects with normal lung parameters; OSA, obstructive sleep apnea; NA, not applicable; HOMA-IR, homeostatic model assessment-insulin resistance; NFS, NAFLD fibrosis score; HFS, Hepamet fibrosis score. Bold values are the statistically significant*.

### Prevalence of NAFLD and Liver Fibrosis

Fatty liver index (FLI) and HSI were calculated and used as an indirect marker of NAFLD in the entire study population, and representative data of each study group are depicted in Table 3. By considering an FLI value equal to or higher than 60 as the optimal cutoff point to identify fatty liver (23), we found that hepatosteatosis was significantly more frequent in patients with OSA (62.1%) than in subjects with NLP (34.9%, *p* = 0.001). Likewise, hepatosteatosis, detected by HSI, was significantly more frequent in patients with OSA (68%) than in subjects with NLP as well (48.8%, *p* = 0.021). Using the OWLiver™ lipidomic test for the non-invasive diagnosis of NAFLD, the prevalence of NASH was significantly higher in patients with OSA (50.3%) than in the NLP group (27.9%, *p* = 0.009) (Table 3).

We also wanted to estimate the presence or absence of advanced fibrosis (F3–F4) in the whole study population by using blood-based non-invasive algorithms such as FIB-4, NFS, and HFS. As depicted in Table 3, only a small proportion of the patients and controls studied had a high risk of advanced liver fibrosis regardless of the fibrosis algorithm used. However, NFS pointed out the highest proportion of individuals with high risk for advanced fibrosis in patients with OSA with respect to the NLP group (6.5 vs. 2.3%, respectively) but with no significant differences between them (*p* = 0.461).

### Risk Factors for Metabolic Disorders

By using univariate and multivariate logistic regression analysis, we found that BMI (OR, 1.25; 95% CI: 1.14–1.37, *p* = 0.001) and an ODI higher than 10 (OR, 2.90; 95% CI: 1.00–8.35, *p* = 0.049) were significantly associated with the risk of insulin resistance (HOMA-IR ≥ 3) in patients with OSA (Supplementary Table 1). On the other hand, a Tc90% higher than 10 was the only variable independently associated with dyslipidemia besides T2D in the cohort of patients with OSA (OR, 2.42; 95% CI: 1.23–4.74, *p* = 0.010) (Supplementary Table 2).

### Risk Factors for NASH

In patients with OSA, male gender (OR, 2.89; 95% CI: 1.32–6.35, *p* = 0.008), increased BMI (OR, 1.12; 95% CI: 1.04–1.20, *p* = 0.003), presence of T2D (OR, 3.50; 95% CI: 1.19–10.28, *p* = 0.022), and a Tc90% ≥10% (OR, 2.32; 95% CI: 1.12–4.81, *p* = 0.023) were significantly associated with a serum lipidomic profile compatible with NASH (Table 4).

**Table 4:** Univariate and multivariate analysis of the independent variables associated with non-alcoholic steatohepatitis (NASH) in patients with obstructive sleep apnea (OSA) (*n* = 153).

**Independent variables**	**Univariate analysis**			**Multivariate analysis**		
	**OR**	**95% CI**	***p*-value**	**OR**	**95% CI**	***p-*value**
Age (years)	1.00	(0.96–1.03)	0.887			
Sex (female/male)	1.82	(0.93–3.56)	0.081	2.89	(1.32–6.35)	**0.008**
BMI (kg/m^2^)	1.12	(1.05–1.20)	<0.001	1.12	(1.04–1.20)	**0.003**
Diabetes (no/yes)	3.82	(1.43–10.20)	0.007	3.50	(1.19–10.28)	**0.022**
AHI (mild/high)	1.65	(0.84–3.24)	0.143			
ODI (low/high)	1.24	(0.52–2.97)	0.631			
Tc90% (low/high)	2.78	(1.44–5.36)	0.002	2.32	*(*1.12–4.81)	**0.023**

*OSA, obstructive sleep apnea; OR, odds ratio; CI, confidence interval; BMI, body mass index; AHI, apnea-hypopnea index; ODI, oxygen desaturation index; Tc90%, percentage of sleep time with oxygen saturation <90%*.

## Discussion

This prospective study provides evidence that obesity, T2D, and metabolic syndrome were more frequent in patients with OSA than in subjects with NLP, but without statistical significance. However, the prevalence of insulin resistance and dyslipidemia was significantly higher in patients with OSA than in subjects with NLP (*p* < 0.001 and *p* < 0.003, respectively). Moreover, by using univariate and multivariate logistic regression analysis, we found that an ODI that was equal to or higher than 10 events/hour was significantly associated with the risk of insulin resistance (OR, 2.90; 95% CI: 1.00–8.35, *p* = 0.049), whereas a Tc90% that was equal or higher than 10% was the only variable independently associated with dyslipidemia in patients with OSA (OR, 2.42; 95% CI: 1.23–4.74, *p* = 0.010).

On the other hand, regarding NAFLD, we observed that hepatosteatosis, by using either FLI or HSI, was significantly more frequent in patients with OSA (62.1 and 68%, respectively) than in subjects with NLP (34.9%, *p* = 0.001, and 48.8%, *p* = 0.021, respectively). In addition, by using the OWLiver™ lipidomic test, we detected a lipidomic profile compatible with NASH in 50.3% of patients with OSA compared with 27.9% in subjects with NLP, with a statistically significant difference (*p* = 0.009). Our findings reinforce the previous evidence provided in a systematic review and meta-analysis conducted by Musso et al. indicating a pathogenic link between OSA and NASH (2). Because metabolomics and lipidomics appear to be useful in identifying biomarkers related to NAFLD and its complications, such as NASH and HCC, among others (32), we are planning to explore the efficacy of the OWLiver™ test to detect new cases of NASH and HCC in a prospective longitudinal study of patients with OSA.

Diagnosis of NASH is clinically relevant as it has been associated with an increased risk of liver-specific mortality, but is the fibrosis stage, however, what actually increases the risk of all-cause mortality and liver-related morbidity in patients with NAFLD (33)? Notably, in the present study, we observed, by using three distinct algorithms to estimate hepatic fibrosis, only a small percentage of patients with OSA (average of 3%) had a high risk of advanced liver fibrosis similar to that found in subjects with NLP (average of 2%), with no significant differences between both groups. These data appear to indicate that patients with OSA are at low risk for advanced liver fibrosis but, given that the major limitation of the distinct fibrosis scores used in this study is that an average of 30% of their values fall in-between the lower and upper thresholds (indeterminate results), interpretation of our findings is intriguing and must be made with caution.

The major novelty of the present study is that according to logistic regression analysis adjusted by confounders, a Tc90% equal or higher than 10% was the only polygraphic variable independently associated with NASH in patients with OSA (OR, 2.32; 95% CI: 1.12–4.81, *p* = 0.023). Because increased Tc90% and ODI are the best markers of nocturnal intermittent hypoxia (34), our findings showed herein suggest that nocturnal intermittent hypoxia could contribute to NASH development and progression in patients with OSA but, although there is increasing scientific evidence indicating that chronic intermittent hypoxia promotes NAFLD and liver fibrosis in rodents (35–37), further experimental studies are needed to fully elucidate this important issue.

In conclusion, this study shows that metabolic comorbidities such as insulin resistance, dyslipidemia, and NASH are highly prevalent in patients with OSA. Interestingly, male gender, increased BMI, presence of T2D, and high Tc90% were the only predictors of NASH in patients with OSA. This study suggests that the OWLiver™ lipidomic test may be useful for the screening of NASH in patients with OSA in order to identify patients at risk for NASH to whom in-depth hepatologic evaluation must be recommended.

## Data Availability Statement

The original contributions presented in the study are included in the article/Supplementary Material, further inquiries can be directed to the corresponding author/s.

## Ethics Statement

The studies involving human participants were reviewed and approved by La Princesa Universitary Hospital's Clinical Research Ethics Committee. The patients/participants provided their written informed consent to participate in this study.

## Author Contributions

PL, JA, ÁG-R, and CG-M conceived and supervised the study. PL, CF-G, BA-O, MH-O, CA-G, and EZ-G recruited patients and were involved in data generation. PL, CF-G, ÁG-R, and CG-M analyzed and discussed data. PL, ÁG-R, and CG-M prepared the manuscript. All authors critically revised and approved the manuscript.

## Funding

This work was supported by grants PI17/00535 and PI20/00837 from the Instituto de Salud Carlos III (ISCIII, Spain) and Fondo Europeo para el Desarrollo Regional (FEDER) to CG-M; Beca SEPAR 2016 (Sociedad Española de Neumología y Cirugía Torácica, Spain) to PL; and grants PI19/00123 from ISCIII/FEDER, Spain, Beca Eduardo Gallego 2016 (Fundación Francisco Cobos, Spain) and CIBERdem (ISCIII) to ÁG-R.

## Conflict of Interest

The authors declare that the research was conducted in the absence of any commercial or financial relationships that could be construed as a potential conflict of interest.

## Publisher's Note

All claims expressed in this article are solely those of the authors and do not necessarily represent those of their affiliated organizations, or those of the publisher, the editors and the reviewers. Any product that may be evaluated in this article, or claim that may be made by its manufacturer, is not guaranteed or endorsed by the publisher.
