# Supplementary material for: Increased Oxygen Desaturation Time During Sleep Is a Risk Factor for NASH in Patients With Obstructive Sleep Apnea: A Prospective Cohort Study
Source: Front Med (Lausanne). 2022 Feb 23;9:808417. doi: 10.3389/fmed.2022.808417 (PMC8906568; doi:10.3389/fmed.2022.808417)
Supplement: Supplementary file 1 [file Table_1.docx]

**Supplementary Table 1. Univariate and multivariate analysis of the independent variables associated with HOMA-IR score ≥ 3 in patients with OSA (n= 153)**

| **Independent variables** | **Univariate analysis** | | | **Multivariate analysis** | | |
| --- | --- | --- | --- | --- | --- | --- |
|  | **OR** | **95% CI** | ***p* value** | **OR** | **95% CI** | ***p* value** |
| Age (years) | 1.01 | [0.98-1.05] | 0.481 |  |  |  |
| Sex (female/male) | 0.77 | [0.39-1.53] | 0.451 |  |  |  |
| BMI (kg/m^2^) | 1.26 | [1.15-1.38] | <0.001 | 1.25 | [1.14-1.37] | **<0.001** |
| T2D (no/yes) | 1.82 | [0.71-4.66] | 0.214 |  |  |  |
| AHI (mild/high) | 1.82 | [0.90-3.68] | 0.098 | 0.96 | [0.41-2.27] | 0.928 |
| ODI (low/high) | 3.91 | [1.55-9.85] | 0.004 | 2.90 | [1.00-8.35] | **0.049** |
| Tc90% (low/high) | 1.26 | [0.65-2.41] | 0.494 |  |  |  |

OSA, obstructive sleep apnea; OR, odds ratio; CI, confidence interval; BMI, body mass index; T2D, type 2 diabetes; AHI, apnea-hypopnea index; ODI, oxygen desaturation index; Tc90%, percentage of sleep time with oxygen saturation less than 90%.
