# Supplementary material for: Increased Oxygen Desaturation Time During Sleep Is a Risk Factor for NASH in Patients With Obstructive Sleep Apnea: A Prospective Cohort Study
Source: Front Med (Lausanne). 2022 Feb 23;9:808417. doi: 10.3389/fmed.2022.808417 (PMC8906568; doi:10.3389/fmed.2022.808417)
Supplement: Supplementary file 2 [file Table_2.docx]

**Supplementary Table 2. Univariate and multivariate analysis of the independent variables associated with dyslipidemia in patients with OSA (n=153)**

| **Independent variables** | **Univariate analysis** | | | **Multivariate analysis** | | |
| --- | --- | --- | --- | --- | --- | --- |
|  | **OR** | **95% CI** | ***p* value** | **OR** | **95% CI** | ***p* value** |
| Age (years) | 1.03 | [0.99-1.07] | 0.113 |  |  |  |
| Sex (female/male) | 0.57 | [0.29-1.11] | 0.100 | 0.58 | [0.29-1.18] | 0.132 |
| BMI (kg/m^2^) | 1.04 | [0.98-1.09] | 0.218 |  |  |  |
| T2D (no/yes) | 3.31 | [1.29-8.47] | 0.013 | 3.27 | [1.24-8.58] | **0.016** |
| AHI (mild/high) | 1.17 | [0.60-2.28] | 0.642 |  |  |  |
| ODI (low/high) | 0.76 | [0.32-1.82] | 0.534 |  |  |  |
| Tc90% (low/high) | 2.37 | [1.24-4.56] | 0.009 | 2.42 | [1.23-4.74] | **0.010** |

OSA, obstructive sleep apnea; OR, odds ratio; CI, confidence interval; BMI, body mass index; T2D, type 2 diabetes; AHI, apnea-hypopnea index; ODI, oxygen desaturation index; Tc90%, percentage of sleep time with oxygen saturation less than 90%.
