# Supplementary figures and images for: Increased Oxygen Desaturation Time During Sleep Is a Risk Factor for NASH in Patients With Obstructive Sleep Apnea: A Prospective Cohort Study
Source: Front Med (Lausanne). 2022 Feb 23;9:808417. doi: 10.3389/fmed.2022.808417 (PMC8906568; doi:10.3389/fmed.2022.808417)

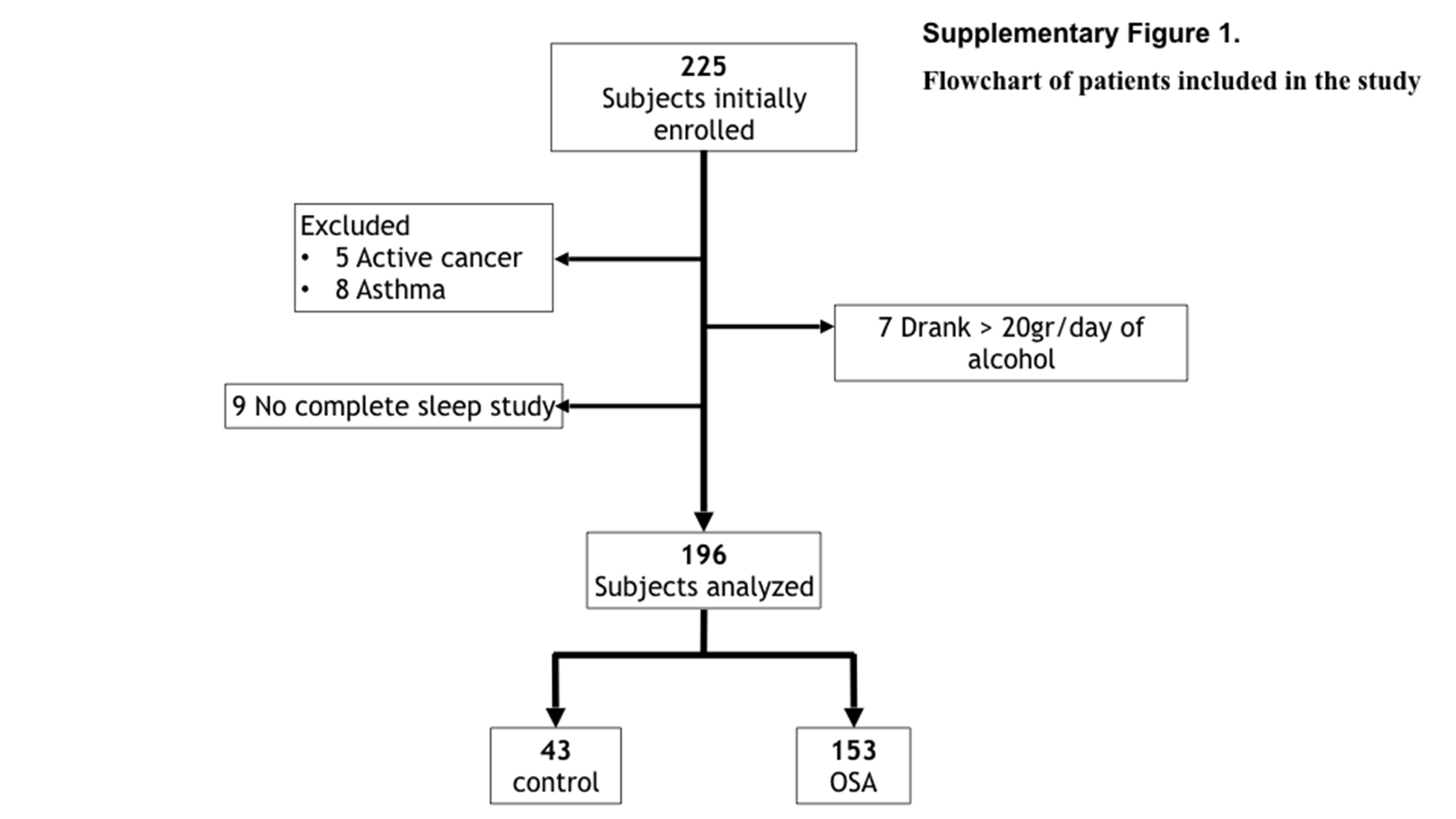

Supplement: Supplementary file 3 [file Image_1.TIF]
